# Mean platelet volume-to-albumin ratio as a predictor of pathological complete response in HER2-positive breast cancer undergoing neoadjuvant chemotherapy

**DOI:** 10.1007/s12672-026-04938-w

**Published:** 2026-04-14

**Authors:** Hatice Ayyıldız Sevim, Mehmetcan Atak, Zeynep Altundağ Derin, Selahattin Çelik, İsmail Burak Gültekin, Doğan Yazılıtaş

**Affiliations:** 1 Department of Medical Oncology, Ankara Etlik City Hospital, Ankara, Türkiye; 2 Department of Gynecology, Ankara Etlik City Hospital, Ankara, Türkiye

**Keywords:** Breast cancer, HER2-positive, MPV/Alb ratio, Neoadjuvant chemotherapy, Pathological complete response

## Abstract

**Objective:**

To evaluate the predictive value of the mean platelet volume–to–albumin (MPV/Alb) ratio for pathological complete response (pCR) in patients with human epidermal growth factor receptor 2 (HER2)-positive breast cancer (BC) treated with neoadjuvant chemotherapy (NACT).

**Methods:**

In this retrospective single-center study, 139 consecutive patients with HER2-positive breast cancer who received dual anti-HER2 therapy combined with anthracycline- and taxane-based neoadjuvant chemotherapy between December 2022 and February 2025 were included. Clinicopathological variables, immuno-inflammatory parameters, and the MPV/Alb ratio were analyzed. Predictors of pCR were assessed using univariable and multivariable logistic regression. Discriminative performance and an optimal cut-off for MPV/Alb were determined by receiver operating characteristic (ROC) analysis.

**Results:**

The median age was 50 years (range, 26–79), and pCR was achieved in 54.7% of patients. On univariable analyses, estrogen receptor (ER) status, progesterone receptor (PR) status, and MPV/Alb were associated with pCR. In multivariable analysis, only MPV/Alb remained an independent predictor of pCR (*p* = 0.012; odds ratio [OR], 0.388; 95% confidence interval [CI], 0.185–0.813). ROC analysis identified an optimal MPV/Alb cutoff of 2.32 (AUC 0.626; 95% CI 0.535–0.720; *p* = 0.048), yielding 64.5% sensitivity and 58.7% specificity.

**Conclusions:**

The MPV/Alb ratio may serve as an independent predictor of pCR in HER2-positive BC undergoing NACT. As a readily obtainable, low-cost composite derived from routine laboratory tests, MPV/Alb could aid risk stratification and treatment decision-making in clinical practice.

## Introduction

 Human epidermal growth factor receptor 2 (HER2)-positive breast cancer (BC) accounts for approximately 15–20% of all breast cancers and is associated with an aggressive clinical course and poor prognosis [1]. Current treatment standards for locally advanced HER2-positive BC favor neoadjuvant chemotherapy (NACT). This approach significantly increases the rate of pathological complete response (pCR), which is closely linked to improved long-term survival [2, 3].

While achieving pCR after NACT is associated with markedly better outcomes, patients who fail to attain pCR or who experience disease progression generally have poorer prognoses [4]. Therefore, accurately predicting NACT response before therapy initiation could guide individualized treatment strategies and optimize outcomes. Several immunologic and histopathologic biomarkers based on blood counts have been proposed for this aim [5, 6]. Among immuno-inflammatory markers based on blood counts, absolute neutrophil (N), platelet (P), monocyte (M), and lymphocyte (L) counts, as well as composite indices such as the neutrophil-to-lymphocyte ratio (NLR), platelet-to-lymphocyte ratio (PLR), and monocyte-to-lymphocyte ratio (MLR), have been evaluated as prognostic factors across various malignancies [7–9]. In HER2-positive breast cancer, the relevance of inflammatory biomarkers is further supported by the distinct immune microenvironment of this subtype. Higher levels of tumor-infiltrating lymphocytes have been associated with increased rates of pathological complete response following neoadjuvant therapy, underscoring the importance of host inflammatory and immune context in treatment sensitivity [10].

MPV is one of these inflammation-based markers and tends to increase during systemic inflammation, reflecting platelet activation and heightened systemic stress [11]. In contrast, serum albumin typically decreases under inflammatory conditions, serving as an indirect marker of nutritional status and systemic stress [12–15]. Collectively, these findings suggest that the MPV/Alb ratio may represent a composite indicator of the inflammatory–nutritional axis in cancer, integrating platelet-related inflammatory activity with nutritional and negative acute-phase status more comprehensively than either parameter alone. Current evidence indicates that both MPV and serum albumin levels possess prognostic relevance in breast cancer [16, 17]. However, the MPV/Alb ratio has been investigated in only a single study within an oncologic setting, wherein lower MPV/Alb values were reported to be associated with increased mortality among patients with febrile neutropenia [18]. HER2-positive breast cancer was specifically selected because this subtype has a distinct immune-inflammatory context, and treatment response may be influenced not only by tumor biology but also by host–tumor microenvironment interactions [19]. Moreover, despite standardized neoadjuvant treatment, marked variability in pCR remains, suggesting that systemic inflammatory and nutritional status may be particularly relevant in this subgroup. Therefore, HER2-positive disease represents a clinically meaningful setting in which to evaluate a composite biomarker such as MPV/Alb. The present study therefore aims to investigate the relationship between the MPV/Alb ratio and pCR in HER2-positive BC patients receiving NACT.

## Methods

### Patient population

In this retrospective study, records of 1,500 patients with BC treated at the Medical Oncology Department of our hospital between December 2022 and February 2025 were reviewed. Among these, 215 patients who received NACT with anti-HER2 therapy and had HER2 amplification histopathologically confirmed on core needle biopsy were evaluated.

Exclusion criteria included missing laboratory or clinical data (*n* = 30), trastuzumab monotherapy without pertuzumab (*n* = 16), and loss to follow-up (*n* = 30). Ultimately, 139 patients with confirmed HER2 amplification (Immunohistochemistry (IHC) 2+/3 + and Fluorescence In Situ Hybridization (FISH)-positive) were included in the final analysis.

Inclusion criteria were non-metastatic disease and adequate hepatic, renal, hematologic, and cardiac function for planned neoadjuvant treatment. Adequate organ function was defined as AST and ALT ≤ 2.5 times the upper limit of normal, total bilirubin ≤ 1.5 times the upper limit of normal, creatinine clearance ≥ 50 mL/min, absolute neutrophil count ≥ 1,500/mm³, platelet count ≥ 100,000/mm³, hemoglobin ≥ 10 g/dL, and baseline left ventricular ejection fraction (LVEF) ≥ 50%. Patients with prior malignancies, active infections, or comorbidities compromising treatment tolerance were excluded.

### Laboratory parameters

Baseline complete blood count and biochemistry parameters obtained at treatment initiation—including albumin, lymphocyte and platelet counts—were recorded together with immune-inflammatory indices and the MPV/Alb ratio to assess their association with pathological response. Parameters were calculated as follows:

- NLR: absolute neutrophil count / absolute lymphocyte count.
- PLR: platelet count / absolute lymphocyte count.
- MLR: monocyte count / absolute lymphocyte count.
- MPV/Alb: mean platelet volume (MPV) / serum albumin (Alb).

Peripheral blood samples were obtained under fasting conditions in the morning at treatment initiation, as part of routine pretreatment laboratory testing. Because of the retrospective design, the exact interval between blood collection and laboratory analysis could not be uniformly retrieved from the medical records. MPV was measured using an automated Sysmex hematology analyzer, and serum albumin was measured on the Roche cobas c702 module as part of routine pretreatment laboratory assessment. All measurements were performed according to the laboratory’s standard operating procedures and routine quality control practices.

Pathological complete response was defined as the absence of invasive tumor cells in both the breast and axillary lymph nodes (ypT0/is, ypN0). Accordingly, residual ductal carcinoma in situ alone (ypTis) was not considered to preclude pCR.

Body mass index (BMI) is calculated by dividing a person’s body weight in kilograms by the square of their height in meters. In our study, a BMI ≤ 24 kg/m² was classified as “normal weight,” while a BMI > 24 kg/m² was defined as “overweight/obese.”

### Treatment

All patients received an anthracycline- and taxane-based NACT. Doxorubicin (60 mg/m²) plus cyclophosphamide (600 mg/m²) were administered for four cycles, followed by docetaxel (75 mg/m²) every three weeks. Dual HER2 blockade was initiated during the taxane phase with trastuzumab (8 mg/kg loading, 6 mg/kg maintenance) and pertuzumab (840 mg loading, 420 mg maintenance) every three weeks. Treatment duration ranged from 18 to 24 weeks.

Cardiac function was evaluated via echocardiography every three months. Following completion of therapy, all patients underwent surgery, and pCR status was pathologically determined.

### Statistical analysis

Data analyses were conducted using SPSS version 26.0 (IBM Corp., Armonk, NY). The Kolmogorov–Smirnov test assessed normality. Continuous variables are expressed as mean ± SD or median (range), and categorical variables as counts and percentages. Comparisons between pCR and non-pCR groups employed chi-square or Fisher’s exact tests for categorical variables, and t-tests or Mann–Whitney U tests for continuous variables.

Univariate and multivariate logistic regression analyses identified independent predictors of pCR. Receiver operating characteristic (ROC) analysis was used to evaluate the discriminative ability of MPV/Alb, reporting AUC, 95% confidence interval (CI), sensitivity, and specificity. A p-value < 0.05 was considered statistically significant.

## Results

### Clinicopathological characteristics

A total of 139 women met the eligibility criteria and were included in the analysis. The median age was 50 years (range, 26–79). A total of 108 patients (77.7%) were older than 40 years. Seventy-three patients (52.5%) were premenopausal. Patient characteristics are summarized in Table 1.

**Table 1:** Clinicopathological characteristics of the patients (*n* = 139)

Variable	*n* (%)/median (min–max)
Age (years)	Median 50 (26–79)
< 40	31 (22.3)
≥ 40	108 (77.7)
Diabetes mellitus	
Present	16 (11.5)
Absent	123 (88.5)
Hypertension	
Present	28 (20.1)
Absent	111 (79.9)
Menopausal status	
Premenopausal	73 (52.5)
Postmenopausal	66 (47.5)
ER status	
Positive	85 (61.2)
Negative	54 (38.8)
PR status	
Positive	64 (46.0)
Negative	75 (54.0)
Ki-67	
< 20%	11 (7.9)
≥ 20%	128 (92.1)
Grade	
Grade 1–2	65 (46.8)
Grade 3	74 (53.2)
N stage	
N0	33 (23.7)
N1	89 (64.0)
N2	15 (10.8)
N3	2 (1.5)
T stage	
T1–T2	116 (83.4)
T3–T4	23 (16.6)
Clinical stage	
Stage I	2 (1.4)
Stage II	106 (76.3)
Stage III	31 (22.3)
Laterality	
Right	68 (48.9)
Left	71 (51.1)
Lymphocyte (×10⁶/L)	2120 (880–4100)
Hb (g/dL)	13.4 (8.3–15.9)
PLR	138.4 (65–330)
NLR	2.1 (0.66–7.96)
MLR	0.23 (0.12–0.78)
MPV (fL)	10.50 (7.60–13.60)
Albumin (g/dL)	4.50 (3.80–5.00)
MPV/Alb ratio	2.33 (1.62–3.16)

ER: estrogen receptor; PR: progesterone receptor; NLR: neutrophil-to-lymphocyte ratio; PLR: platelet-to-lymphocyte ratio; MLR: monocyte-to-lymphocyte ratio

**Table 2:** Association between pathological complete response and clinicopathological characteristics

Variable	pCR− (*n* = 63)	pCR+ (*n* = 76)	*p* value
Age			0.667
< 40 years	13 (20.6)	18 (23.7)	
≥ 40 years	50 (79.4)	58 (76.3)	
Diabetes mellitus			0.690
Absent	55 (87.3)	68 (89.5)	
Present	8 (12.7)	8 (10.5)	
Hypertension			0.160
Absent	47 (74.6)	64 (84.2)	
Present	16 (25.4)	12 (15.8)	
BMI			0.218
≤ 24	14 (22.2)	24 (31.6)	
> 24	49 (77.8)	52 (68.4)	
ER status			0.003
Negative	16 (25.4)	38 (50.0)	
Positive	47 (74.6)	38 (50.0)	
PR status			< 0.001
Negative	23 (36.5)	52 (68.4)	
Positive	40 (63.5)	24 (31.6)	
Ki-67			0.993
< 20%	5 (7.9)	6 (7.9)	
≥ 20%	58 (92.1)	70 (92.1)	
Menopausal status			0.163
Premenopausal	29 (46.0)	44 (57.9)	
Postmenopausal	34 (54.0)	32 (42.1)	
Grade			0.875
Grade 1–2	29 (46.0)	36 (47.4)	
Grade 3	34 (54.0)	40 (52.6)	
Laterality			0.780
Right	30 (47.6)	38 (50.0)	
Left	33 (52.4)	38 (50.0)	
T stage			0.514
T1–T2	54 (85.7)	62 (81.6)	
T3–T4	9 (14.3)	14 (18.4)	
N status			0.413
Negative	17 (27.0)	16 (21.1)	
Positive	46 (73.0)	60 (78.9)	
Clinical stage			0.667
Stage I–II	50 (79.4)	58 (76.3)	
Stage III	13 (20.6)	18 (23.7)	
MPV/Alb			0.006
≤ 2.32	37 (58.7)	27 (35.5)	
> 2.32	26 (41.3)	49 (64.5)	
NLR			0.439
≤ 2.13	29 (46.0)	40 (52.6)	
> 2.13	34 (54.0)	36 (47.4)	
PLR			0.804
≤ 138	31 (49.2)	39 (51.3)	
> 138	32 (50.8)	37 (48.7)	

ER: estrogen receptor; PR: progesterone receptor; NLR: neutrophil-to-lymphocyte ratio; PLR: platelet-to-lymphocyte ratio; MLR: monocyte-to-lymphocyte ratio; MPV: mean platelet volume; Alb: albumin; BMI: body mass index

In univariable comparisons, ER status, PR status, and MPV/Alb differed significantly between the pCR and non-pCR groups (*p* = 0.003; *p* < 0.001; *p* = 0.006, respectively). pCR rates were higher in ER-negative and PR-negative tumors, and were also higher among patients with MPV/Alb > 2.32. No significant associations were observed for age (*p* = 0.667), diabetes mellitus (*p* = 0.690), hypertension (*p* = 0.160), BMI (*p* = 0.218), menopausal status (*p* = 0.163), grade (*p* = 0.875), laterality (*p* = 0.780), T stage (*p* = 0.514), N stage (*p* = 0.413), clinical stage (*p* = 0.667), NLR (*p* = 0.439), or PLR (*p* = 0.804) Table 2. 

**Table 3:** Factors associated with pathological complete response (pCR) on logistic regression

Variable	B	SE	OR (95% CI)	*p* value
ER status	0.589	0.503	1.802 (0.672–4.833)	0.242
PR status	0.903	0.476	2.476 (0.970–6.271)	0.058
MPV/Alb	−0.947	0.378	0.388 (0.185–0.813)	0.012

ER: estrogen receptor; PR: progesterone receptor; MPV: mean platelet volume; Alb: albumin; SE: standard error; OR: odds ratio; CI: confidence interval

In the multivariable logistic regression, only MPV/Alb remained independently associated with pCR (*p* = 0.012; OR, 0.388; 95% CI, 0.185–0.813), as shown in Table 3. No significant associations with pCR were observed for ER or PR status.

**Fig. 1:**
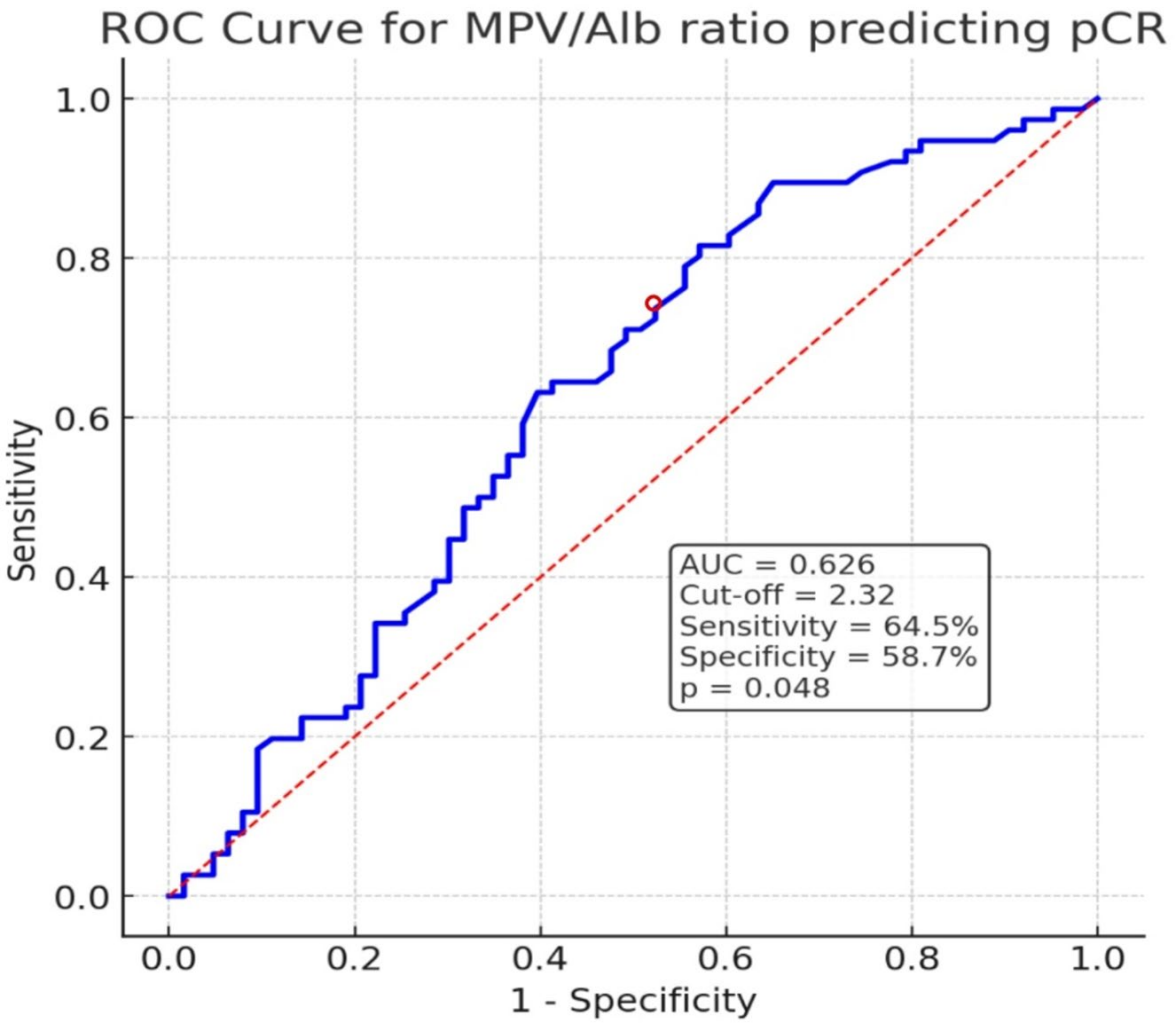
ROC curve of the MPV/Alb ratio for pCR. The optimal cutoff value (2.32) was determined using the Youden index

ROC analysis was performed to evaluate the discriminative ability of MPV/Alb for predicting pCR. The area under the curve (AUC) was 0.626 (95% CI, 0.535–0.720), indicating statistically significant performance (*p* = 0.048). Using an optimal cut-off of 2.32, the sensitivity and specificity were 64.5% and 58.7%, respectively Fig. 1.

**Table 4:** Association between clinicopathological characteristics and the MPV/Alb ratio

Variable	MPV/Alb ≤ 2.32 (*n* = 64)	MPV/Alb > 2.32 (*n* = 75)	*p* value
Age			0.911
< 40 years	14 (21.9)	17 (22.7)	
≥ 40 years	50 (78.1)	58 (77.3)	
Menopausal status			0.636
Premenopausal	35 (54.7)	38 (50.7)	
Postmenopausal	29 (45.3)	37 (49.3)	
ER status			0.691
Negative	26 (40.6)	28 (37.3)	
Positive	38 (59.4)	47 (62.7)	
PR status			0.122
Negative	30 (46.9)	45 (60.0)	
Positive	34 (53.1)	30 (40.0)	
Ki-67			0.555
< 20%	6 (9.4)	5 (6.7)	
≥ 20%	58 (90.6)	70 (93.3)	
Grade			0.715
Grade 1–2	31 (48.4)	34 (45.3)	
Grade 3	33 (51.6)	41 (54.7)	
Laterality			0.656
Right	30 (46.9)	38 (50.7)	
Left	34 (53.1)	37 (49.3)	
BMI			0.848
≤ 24	18 (28.1)	20 (26.7)	
> 24	46 (71.9)	55 (73.3)	
PLR			0.014
≤ 138	25 (39.1)	45 (60.0)	
> 138	39 (60.9)	30 (40.0)	
NLR			0.793
≤ 2.13	31 (48.4)	38 (50.7)	
> 2.13	33 (51.6)	37 (49.3)	

ER: estrogen receptor; PR: progesterone receptor; NLR: neutrophil-to-lymphocyte ratio; PLR: platelet-to-lymphocyte ratio; MPV: mean platelet volume; Alb: albumin; BMI: body mass index

Patients with MPV/Alb ≤ 2.32 had significantly higher PLR levels (*p* = 0.014). No statistically significant associations were observed between MPV/Alb and other clinicopathological features, including age (*p* = 0.911), menopausal status (*p* = 0.636), ER (*p* = 0.691), PR (*p* = 0.122), Ki-67 (*p* = 0.555), grade (*p* = 0.715), laterality (*p* = 0.656), BMI (*p* = 0.848), or NLR (*p* = 0.793), as shown in Table 4.

## Discussion

This study demonstrates that the MPV/Alb ratio serves as a significant and independent predictor of pCR in patients with HER2-positive BC treated with NACT. To our knowledge, this is the first investigation to evaluate the predictive value of MPV/Alb in this specific patient population, integrating both inflammatory and nutritional parameters into a single, easily measurable biomarker.

The overall pCR rate observed in our cohort (54.7%) aligns closely with those reported in previous pivotal studies assessing trastuzumab and pertuzumab–based regimens, in which pCR rates range from 50% to 60% [2, 20]. As expected, hormone receptor (HR)-negative tumors exhibited higher pCR rates compared with HR-positive counterparts, consistent with the findings of Cortazar et al., who demonstrated that pCR is a strong surrogate endpoint for long-term survival, particularly in HR-negative and HER2-positive subgroups [21, 22].

The biological rationale linking the MPV/Alb ratio to therapeutic response is multifaceted. MPV reflects platelet activation and turnover, both of which are closely intertwined with systemic inflammation and tumor progression [23]. Larger, metabolically active platelets release pro-tumorigenic cytokines, chemokines, and growth factors such as VEGF and PDGF, promoting angiogenesis, invasion, and resistance to therapy [24, 25]. On the other hand, serum albumin is a negative acute-phase reactant that decreases during chronic inflammation or malnutrition, representing both the nutritional and inflammatory status of the patient [26, 27]. Low serum albumin levels have been consistently associated with poor outcomes and impaired chemotherapy tolerance in several malignancies, including breast cancer [28]. The combination of these two parameters into a ratio (MPV/Alb) thus provides a more integrated measure of host–tumor interactions and overall systemic stress.

In the present study, patients with an MPV/Alb ratio above 2.32 achieved significantly higher pCR rates, suggesting that a more favorable inflammatory–nutritional balance may enhance chemosensitivity. This finding resonates with prior evidence linking systemic inflammation indices such as the neutrophil-to-lymphocyte ratio (NLR) and platelet-to-lymphocyte ratio (PLR) with treatment outcomes in breast cancer [7–9]. Interestingly, we observed an inverse relationship between MPV/Alb and PLR, implying that the MPV/Alb ratio may capture distinct yet complementary aspects of the systemic inflammatory response. Our data also align with studies reporting the dual role of platelets in cancer biology. Elevated MPV has been variably associated with either poor or favorable prognosis depending on tumor type, disease stage, and treatment setting [17, 29, 30]. These discrepancies underscore the complexity of platelet dynamics in the tumor microenvironment. The MPV/Alb ratio, by incorporating both platelet activity and nutritional status, may overcome some of these inconsistencies and serve as a more stable biomarker reflecting systemic homeostasis.

An additional finding of our study was the significant association between MPV/Alb and PLR. This observation is biologically plausible, as both indices are linked to systemic inflammatory activity and platelet-related immune processes. However, although PLR was significantly associated with MPV/Alb, it was not significantly associated with pCR in our cohort, whereas MPV/Alb remained independently associated with pCR in the multivariable analysis. This discrepancy may suggest that MPV/Alb reflects a broader host-related biological profile by integrating platelet activation with nutritional and inflammatory status, while PLR represents a narrower inflammatory dimension. Accordingly, MPV/Alb may provide complementary predictive information beyond that offered by conventional hematological inflammatory indices alone.

From a clinical perspective, the MPV/Alb ratio offers several practical advantages. It is inexpensive, based on routinely available laboratory parameters, non-invasive, and can be readily incorporated into standard pretreatment assessment. In resource-limited settings where access to molecular profiling may be restricted, this biomarker may help identify patients at higher risk of an inadequate response to neoadjuvant therapy, thereby supporting early treatment optimization or referral to treatment-intensification strategies. Although molecular and mutation-based predictive tools are becoming increasingly important in breast cancer within the framework of precision oncology, readily accessible hematological biomarkers such as MPV/Alb may still provide complementary and pragmatic information, particularly in settings where advanced molecular testing is not universally available [31].

The fact that this index has not been previously evaluated in malignancy and that our study is the first to investigate this issue constitutes one of the major strengths of our study. Nevertheless, this study has limitations that merit consideration. The retrospective, single-center design inherently carries risks of selection bias and unmeasured confounding. In addition, potential inter-center differences in diagnostic practices, treatment protocols, and laboratory procedures were not captured, which may limit the external generalizability of the findings. Although the sample size was comparable to that of other biomarker-based exploratory analyses, it may have further restricted the generalizability of the results. External validation in larger, multi-institutional cohorts and the integration of MPV/Alb with emerging molecular predictors such as tumor-infiltrating lymphocytes or circulating tumor DNA could further refine its predictive value.

In conclusion, our findings highlight the MPV/Alb ratio as a novel, independent, and easily accessible biomarker for predicting pCR in patients with HER2-positive BC receiving NACT. By integrating inflammatory and nutritional parameters, this index provides a comprehensive reflection of host–tumor interactions and systemic response. Future prospective and multicenter studies are warranted to validate these results and to explore its potential role in guiding individualized treatment strategies.

## Data Availability

The datasets generated or analyzed during the current study are available from the corresponding author on reasonable request.
